# Supplementary material for: Are carbon emissions trading and green financial instruments synergistic? -Comprehensive quantitative research based on content analysis
Source: PLoS One. 2024 Mar 7;19(3):e0298601. doi: 10.1371/journal.pone.0298601 (PMC10919601; doi:10.1371/journal.pone.0298601)
Supplement: S1 Data — (ZIP) [file pone.0298601.s001.zip › Data/地级市Z_F工作报告.zip!/╣π╬≈/╙±┴╓╩╨/└┤╫╘╣1⁄2╓┌║┼í╛┬φ┐╦╩2╛▌═°í┐_2019.docx]

各位代表：

现在，我代表市人民政府向大会作政府工作报告，请予审议，并请市政协委员和列席会议的同志提出意见。

一、2018年工作回顾

2018年是极不平凡的一年。面对年初企业生产经营困难、工业投资持续低迷、环境治理难度加大等严峻挑战，在自治区党委、政府以及市委的坚强领导下，全市人民迎难而上，担当作为，真抓实干，奋勇拼搏，有效应对各种困难挑战，奋力完成各项目标任务，夺取了改革发展的新胜利，在实现“两个建成”的征程中迈出了新的坚实步伐。

——经济实力跃上新台阶。初步统计，GDP增长7.2%，第一产业增加值增长5.3%，规模以上工业增加值增长5.8%，财政收入增长6.9%，固定资产投资增长14.7%，社会消费品零售总额增长10.6%。

——县域发展开创新局面。北流市、容县、玉州区连续两年获评为广西科学发展先进县（城区），是全区获奖最多的市之一，玉州区县域经济发展的成功经验获自治区政府通报表扬。全市7个县（市、区）全部进入全域旅游示范区创建单位，北流市成功创建广西特色旅游名县。玉州区、容县获评为全区健康产业示范县。兴业县获评为“广西氧化钙之乡”。

——千亿产业迈出新步伐。玉柴上划自治区管理，获得自治区全面支持，有效化解了债务风险，引进了一批配套项目，“二次创业”取得突破；广西先进装备制造城（玉林）建设全面推进，引进项目22个，新开工10个，总投资58亿元。玉林（福绵）节能环保产业园、生态纺织服装产业园建设加快推进，落户企业139家，中滔环保集团总部决定搬迁玉林，整建制承接产业转移模式成为全区招商典范。总投资56亿元的柳钢中金500万吨不锈钢基地（一期）开工建设，成为柳钢集团三大基地之一。中医药健康产业园大参林药业（一期）、康臣玉药等项目加快推进。机械制造、服装皮革、新材料、大健康四大千亿产业加速形成。

——交通建设实现新突破。经过几届市四家班子领导、各级各部门和全市人民的长期不懈努力，时速350公里的南宁至玉林城际铁路建设拉开序幕，玉林人民的高铁梦走向现实！机场大道全线贯通，玉林福绵机场建设进展顺利。铁山港东岸2个10万吨级码头开工建设，向海发展取得新进展。浦北至北流清湾高速公路开工建设，玉林至湛江、荔浦至玉林、松旺至铁山港东岸高速公路加快推进，“两横四纵”高速公路网主骨架初步形成。

——环境治理取得新成果。空气质量成功扭转年初重度污染的被动局面，优良天数比例完成自治区考核目标，PM2.5浓度同比下降2.5%。南流江水质从1～5月劣Ⅴ类迅速扭转至下半年有5个月达到Ⅲ类，实现年均Ⅳ类水、基本消除支流劣Ⅴ类水体目标，完成了几乎不可能完成的任务。九洲江水质连续三年均值稳定达到Ⅲ类，成为跨省区中小流域水环境治理典范。我市创新畜禽养殖污染治理模式得到国务院第五次大督查通报表扬。

——民生保障得到新改善。脱贫攻坚取得决定性进展，实现8万多贫困人口脱贫、132个贫困村摘帽。广西医科大学玉林校区（一期）、市骨科医院新院落成启用，市图书馆新馆、市颐养园（一期）等一批重大民生项目竣工。玉林往返广州实现动车快速换乘。全市民生支出298亿元，占一般公共预算支出的81%。居民人均可支配收入增长7.1%，居民消费价格涨幅控制在3%左右，人民群众的获得感、幸福感全面提升。

一年来，我们坚持以习近平新时代中国特色社会主义思想为指导，全面贯彻党的十九大和十九届二中、三中全会精神，贯彻落实中央、自治区和市委的决策部署，坚持稳中求进工作总基调，落实高质量发展要求，以供给侧结构性改革为主线，统筹稳增长、促改革、调结构、惠民生、防风险，主要抓了以下工作：

（一）聚焦重大项目建设，积蓄经济发展后劲。实施重大项目建设攻坚，落实领导联系重大项目责任制，建立红黑榜制度，全力促进重大项目落地做成。开工重大项目268个、竣工114个，累计完成投资659.3亿元。远景北流风电、三环高性能陶瓷复合透水砖、博世科固废资源化利用、菱通集成电路板、西陇化工、红狮水泥、诚钢碳酸钙等项目竣工投产，3D打印、金特安特种轮胎、玉柴新能源汽车、岭南中药配方颗粒等项目加快推进。招商引资工作取得新突破，引进柳钢集团、正邦集团、上海电汽集团、长源东谷等企业重大项目99个，总投资665.4亿元。

（二）聚焦重点产业发展，促进新旧动能转换。大力推动工业高质量发展，新增规模以上工业企业54家，新材料产业增长22.1%，大健康产业增长15%。玉柴研发生产的国内首台国六发动机通过认证，实现从国一至国六排放标准升级的全程领跑。推动服务业提档升级，万达广场、奥园广场、金城广场、北流亿联建材家居五金城等一批商贸物流项目稳步推进；宏进农产品批发市场、中药材专业市场获批为农业农村部定点市场。银行机构存贷款余额分别增长10.4%和17.2%。铜石岭、大容山、鹿峰山、六万山、都峤山、本草特色小镇等39个重点旅游项目加快推进，完成旅游投资23.5亿元。旅游接待总人数、旅游总消费分别增长31.4%和38.6%。茂林镇通过国际慢城总部验收。大力推进质量强玉和品牌建设，表彰奖励一批获得市长质量奖、广西名牌等称号的企业。圆满举办第十届药博会，荣膺“改革开放40年·中国优秀会展城市”称号。

（三）聚焦乡村振兴战略，推动城乡协调发展。规划建设乡村振兴项目538个，总投资140亿元。镇镇通二级公路、“四好农村路”建设加快推进，新改建农村公路131公里。加快发展现代特色农业，新增广西现代特色农业核心示范区9个，玉州区、陆川县分别获授予玉林三黄鸡、陆川猪中国特色农产品优势区，容县获授予沙田柚广西特色农产品优势区。新认证国家地理标志农产品1个、地理标志证明商标1件、绿色食品3个、无公害农产品48个，玉林农业科技园获评为国家级农业科技园区，“五彩田园”成功创建首批国家农业产业融合发展示范园，北流市六行村、博白县石柳村获认定为第八批全国一村一品示范村。全面完成“美丽玉林·宜居乡村”建设任务，完成改厨改厕17.7万户，玉州区鹤林村、福绵区十丈村等9个行政村获评为“美丽广西”乡村建设示范村，玉州区南流屯、博白县竹子麓屯等31个村屯获评为绿色村屯。玉州区硃砂垌获评为中国历史文化名村。

（四）聚焦大城市建设，推进新型城镇化。加快玉北同城化，大力推进环城交通、重大市政道路、城市新区开发等十大城建工程，推动城市扩容提质，中心城区建成区面积扩大到74.3平方公里。高铁新城、城北中央商务区、玉东湖、玉东文体路生态城等城市片区开发加快推进，民主南路、园博园至五彩田园公路、二环南路扩建、二环西路、新民路等路网改造升级项目快速推进。深入开展全国文明城市、国家卫生城市、广西宜居城市、广西食品安全示范城市“四城同创”工作，城市影响力明显提升。加快城镇化体系建设，促进各县（市、区）城镇特色发展，全市城镇化率提高到48.2%。茂林镇鹿塘社区成为首批“全国农村幸福社区建设示范单位”。

（五）聚焦深化改革开放，激发社会发展活力。开展优化营商环境集中行动，深化“放管服”改革，市本级“一事通办”事项比例居全区前列。深化商事制度改革，大力支持民营经济发展，新增市场主体5.8万户，总量达26.3万户，增幅和总量均居全区前列。实施减税降费和降低企业用电、用水、用气、物流成本举措，共减轻企业负担47.9亿元。全国农村改革试验区工作稳步推进，北流市农村集体经营性建设用地入市规模居全国33个改革试点地区前列。国企国资改革不断深化。国有林场改革通过自治区验收。陆川县农村信用社成功改制农村商业银行。实现全市信用乡镇、信用村“三农”金融服务室全覆盖。大力推进“南向”“东融”，主动融入国家“一带一路”、西部陆海新通道、北部湾经济区、粤港澳大湾区，龙潭产业园、玉港合作园建设加快推进，向海发展格局逐步形成。

（六）聚焦生态环境治理，建设美丽宜居玉林。建立完善环保工作机制，全面加强生态环境保护，城乡人居环境显著改善。打好蓝天保卫战，开展大气污染防治百日攻坚，大力推进城市扬尘综合治理、“散乱污”整治、烟花爆竹禁燃限放、城市垃圾焚烧和农村秸秆焚烧治理行动，空气质量稳步提高。打好碧水保卫战，全面落实河长制，实施“一河一策”，扎实开展重点流域水环境治理；将南流江水环境整治作为首要任务、头号工程，筹措财政资金13.2亿元全面推进畜禽养殖污染、生活污染和工业污染防治，南流江水质稳步好转；市级饮用水水源地67个问题全部完成整改。打好净土保卫战，扎实推进土壤污染防治，严格管控土壤环境风险。抓好中央环境保护督察反馈问题整改，中央第五环境保护督察组“回头看”交办群众举报件全部办理完毕。全市党政机关、学校、医院等公共机构节能减排工作深入开展。

（七）聚焦精准脱贫攻坚，增进社会民生福祉。深入实施脱贫攻坚三年行动计划，建成100个产业扶贫示范基地和100个培训基地，创建就业扶贫车间48家，324个贫困村集体经济收入达到3万元以上。易地扶贫搬迁、教育扶贫、健康扶贫全面推进。加大稳就业力度，城镇登记失业率控制在4%以内。大力支持创新创业，认定创业孵化基地17个，累计入驻企业317家。开展保障农民工工资支付专项整治行动，农民工工资得到有效保障。深入实施学校建设提升工程，新建成学校24所，新增学位2.8万个。义务教育均衡发展水平不断提高，陆川县、兴业县通过国家评估认定，博白县通过自治区督导评估。高考成绩再创新高，一本上线人数、上线率继续位居广西前列。玉林师范学院申硕攻坚取得重大进展，专任教师中博士突破200人。医药卫生体制改革取得新突破，玉林市、北流市、容县公立医院改革获得自治区表彰。投入5.4亿元，实施 105个基层医疗卫生机构能力建设项目。容县获评为全国妇幼健康优质示范县。文化体育事业加快发展，王力博物馆、广西华侨博物馆等公共文化项目稳步推进。媒体融合发展扎实有效。成功举办第四届市运会。加大社保扩面征缴力度，推进社保“一卡通”，社保服务标准化试点通过国家评估。建立职工大额补充医疗费用统筹制度，推进城乡居民基本医疗保险二次报销改革，不断减轻群众医疗负担。住房公积金管理改革通过国家“双贯标”验收，办事效率大幅提高。

（八）聚焦风险防范化解，维护社会和谐稳定。开展金融领域风险排查专项行动，严厉打击非法金融活动，守住不发生系统性金融风险底线。推进信访工作“三化”建设，开展精准排查、精细化解信访积案攻坚活动，有效化解一批信访积案。持续开展扫黑除恶、反恐防恐、禁毒整治，严厉打击各类刑事犯罪，群众安全感和满意度不断提高。深入实施“七五”普法规划。加强食品药品监管，没有发生系统性食品药品安全事件。抗击台风“山竹”等自然灾害取得全面胜利，防灾减灾救灾工作扎实有效。持续开展火灾隐患专项治理，全市没有发生较大以上火灾。全面开展“强监管、严执法”、落实企业安全生产主体责任等专项行动，加强重点行业重点领域专项整治，安全生产形势持续稳定好转。

同时，全面建成16个自治区成立60周年项目，举办系列庆祝活动，民族团结的大好局面进一步巩固。成功举办2018年海外华侨华人玉林恳亲大会，进一步凝聚海内外力量推动玉林发展。创新督促落实机制，抓好国务院第五次大督查移交问题整改，督查工作获得自治区政府3项通报表扬，为全区最多。气象服务、人防建设得到加强，获评为全国防震减灾工作先进单位。着力关爱“三留守”人员，妇女儿童、老年人、未成年人和残疾人权益得到充分保障。社会救助制度日趋完善。宗教、外事、侨务和港澳台事务取得新成效，统计、供销、水库移民、水文、二轻、无线电、方志、档案、机构编制、科普、驻外机构、保密等工作取得新成绩，双拥共建、军民融合工作迈出新步伐，各项社会事业取得新进步。

一年来，我们落实全面从严治党要求，不断加强政府自身建设。深入学习贯彻习近平新时代中国特色社会主义思想，强化“四个意识”，坚定“四个自信”，坚决做到“两个维护”。狠抓中央、自治区和市委各项决策部署的贯彻落实，确保政令畅通、落地见效。坚持向市委、市人大及其常委会报告工作，向市政协通报情况，自觉接受监督，人大代表建议和政协委员提案全部办结。坚持依法行政、依法治市，努力建设法治政府。严格执行“三重一大”集体决策制度。有效推进公平竞争审查制度。深化政务公开和政府信息公开。加强行政绩效管理体系建设，全面提高政府效能。加大整治庸政懒政怠政力度，坚决纠正不作为、慢作为、乱作为。加强重点领域的审计监督和行政监察，财政资金、国有资产、政府采购、公共工程建设、公共资源交易得到有效监管。坚持节用裕民，“三公”经费只减不增。严肃查处违纪违法行为，坚决整治扶贫领域、群众身边的“四风”和腐败问题，努力打造为民务实清廉政府。

各位代表！回顾过去一年，我市改革发展所取得的成绩来之不易、难能可贵。这是自治区党委、政府和市委坚强领导的结果，是市人大及其常委会依法监督的结果，是市政协民主监督和社会各界监督支持的结果，是全市人民团结拼搏、努力奋斗的结果。在此，我代表市人民政府，向全市人民，向人大代表、政协委员，向中央及自治区直属驻玉单位、驻玉部队、武警官兵，向各民主党派、工商联、无党派人士、各人民团体，向海内外所有关心、支持玉林发展的各界人士，表示崇高的敬意和衷心的感谢！

安不忘危，兴不忘忧。我们清醒地看到，困难和问题不容忽视。稳增长的基础不够牢固，经济下行压力依然较大；工业转型升级步伐不够快，发展动能接续不足；土地后备资源匮乏，项目落地难、企业融资难等问题仍然是制约发展的突出瓶颈；生态环境治理难度大，任务艰巨；社会事业发展滞后，民生保障有待加强；营商环境还不够优化，服务发展的质量和效率还有待提高，等等。对这些问题，我们必须高度重视，采取有力措施，切实加以解决。

二、2019年工作思路和主要目标

2019年是新中国成立70周年，是决胜全面建成小康社会第一个百年奋斗目标的关键之年。当前，我国发展仍处于并将长期处于重要战略机遇期，中央加强宏观政策统筹协调，谋划出台系列利好政策，推进稳就业、稳金融、稳外贸、稳外资、稳投资、稳预期，为我们更好推动经济社会发展指明了方向、提振了信心。自治区全面落实“三大定位”新使命和“五个扎实”新要求，全面加快“建设壮美广西、共圆复兴梦想 ”步伐，为我们带来了重大发展机遇。我市四大千亿产业加快形成，一批重大项目落地建设，重大交通基础设施不断完善，为我们加快发展提供了强有力支撑。我们要努力抓住难得的大好机遇，主动作为，创新进取，推动玉林加快发展、跨越发展。

今年工作的总体要求是：以习近平新时代中国特色社会主义思想为指导，全面贯彻党的十九大和十九届二中、三中全会精神，贯彻落实中央、自治区和市委的决策部署，统筹推进“五位一体”总体布局和协调推进“四个全面”战略布局，坚持稳中求进工作总基调，坚持新发展理念，坚持推进高质量发展，坚持以供给侧结构性改革为主线，坚持深化市场化改革、扩大高水平开放，按照“建设壮美广西、共圆复兴梦想”的总目标总要求，着力推进交通基础设施建设，着力推动产业优化升级，着力深化改革开放，着力推进城乡协调发展，着力优化营商环境，着力加强生态环境治理，着力发展壮大民营经济，着力抓好脱贫攻坚和民生建设，全力打好“三大会战”，努力实现“六大突破”，促进经济社会持续健康发展和社会大局稳定，为全面建成小康社会打下决定性基础，以优异成绩迎接新中国成立70周年。

经济社会发展主要预期目标是：地区生产总值增长7.2%，规模以上工业增加值增长7%，财政收入增长5%，固定资产投资增长12%，社会消费品零售总额增长10%，外贸进出口总额增长6%，居民人均可支配收入增长7.2%，节能减排降碳和能源消耗总量控制在自治区下达目标以内，居民消费价格涨幅控制在3%左右，城镇登记失业率控制在4%以内。

提出上述目标，考虑了玉林实现“两个建成”的需要，也是玉林赶超跨越的需要，符合我市当前发展阶段的实际，更是全市人民的殷切希望。面对复杂严峻的经济形势，我们要坚定攻坚克难、勇于突破的信心和决心，担当实干，埋头苦干，全面推进思想再解放、改革再深入、开放再扩大、工作再争先、发展再跨越，全面完成各项目标任务，奋力开创新时代玉林发展新局面！

三、全力打好“三大会战”，努力实现“六大突破”

今年，我们要全力打好“三大会战”，努力实现“六大突破”，以“三大会战”的成效夯实我市发展基础，以“六大突破”的成效推动全市经济社会大发展、快发展。

（一）打好交通建设大会战。坚持不懈抓好“建高铁、修机场、造码头”三件大事，着力构建现代综合交通体系，加快建设面向粤港澳大湾区和北部湾城市群的区域性综合交通枢纽，以交通优势重塑发展优势。

加快高速铁路建设。全面推进南宁至玉林城际铁路建设，大力争取南宁经玉林至深圳、张家界经桂林玉林至海口“一横一纵”2条高铁新通道纳入国家中长期发展规划。聚焦进入高铁时代，谋划布局配套设施、产业，培育发展高铁经济。稳步推进北海—玉林、贺州—梧州—玉林城际铁路项目规划建设。

加快机场码头建设。加快机场建设进度，玉林福绵机场年底建成。加快“一港一路一航道”建设，基本建成铁山港东岸2个10万吨级码头泊位。加快推进绣江复航工程前期工作。

加快高速公路建设。玉林至湛江高速公路（广西段）年底建成通车。加快荔浦至玉林、浦北至北流清湾、松旺至铁山港东岸等高速公路建设。力争开工建设南宁经玉林至珠海高速公路（广西段）。做好南宁经博白至湛江、北流至高州（广西段）、平南至信宜（容县段）等高速公路前期工作。

加快城乡公路建设。加快推进玉林至陆川公路改造和容县绕城公路建设项目。抓好陆川清湖至浦北石埇、平南泗灵至兴业高峰、岑溪南渡至陆川等公路建设。全面抓好23个镇镇通二级公路项目，加快“四好农村路”建设，新改建农村公路170公里。

（二）打好污染防治大会战。牢固树立绿水青山就是金山银山理念，全面加强生态环境保护和建设，全面完成水环境质量、空气环境质量、主要污染物减排等环境保护目标任务。

打赢蓝天碧水净土保卫战。着力加强大气污染防治，重拳整治城区烟花爆竹燃放，严格扬尘监管，严禁大面积焚烧秸秆，深化工业污染源治理，促进空气质量持续稳定提升。着力加强水环境综合治理，深入落实“河长制”，抓好重点流域污染防治。坚定不移把南流江流域水环境整治作为首要任务和头号工程，全民参与，全域治理，重典整治，铁腕治污，实现南流江水质年均值达到Ⅲ类。巩固九洲江治理成效，确保跨省界交接断面水质年均值达Ⅲ类、月达标率83%以上。加强饮用水水源地保护，完成县级饮用水水源地问题整改，确保水质稳定达标。着力加强土壤污染防治，促进土壤资源永续利用，实现土壤环境质量有效改善。加快中央环保督察“回头看”和自治区专项督察反馈问题整改，按时完成整改销号。

加强生态环境建设。加强自然资源保护，精心呵护“山水林田湖草”生命共同体，大力建设国家生态文明先行示范区。抓好森林生态系统建设，推动林木经营向森林经营、林业经济向林区经济转型。抓好湿地生态系统建设，提升保护修复水平，实现水常绿、草长青。抓好流域生态系统建设，推进水资源、水生态、水环境、水灾害“四水同治”。抓好田湖生态系统建设，加强面源污染防治，强化生态种养。加强自然保护区保护和管理，着力提升自然保护区生态系统质量。

大力发展生态经济。加强工业园区环保设施建设，推进工业园区废弃物综合利用，继续实施玉林经济开发区生态化改造，积极推进传统工业向生态环保工业转型。积极发展再生资源加工利用产业。大力推进清洁能源项目建设，支持光伏发电等可再生能源有效利用，加快北流隆盛风电场、博白马子岭风电场（三期）等项目建设。大力推广“益生菌+”现代生态养殖模式，力争畜禽规模养殖场生态养殖比重达90%以上。

（三）打好精准脱贫大会战。深入推进打赢脱贫攻坚三年行动，巩固陆川县、兴业县脱贫摘帽成果，实现6万多贫困人口脱贫、90多个贫困村摘帽、博白县脱贫摘帽目标任务。

精准抓好产业扶贫。扎实推进“三个一百”扶贫产业工程，进一步拓宽和深化产业扶贫模式，充分发挥产业扶贫专项资金作用，培育壮大稳定增收产业。建立利益联结机制，全面提高贫困农户发展产业的参与度和组织化程度，实现边缘贫困户产业扶贫全覆盖，深度贫困户再覆盖。大力发展村级集体经济，实现所有贫困村集体经济年收入达到4万元。

精准抓好设施建设。打造一批贫困村提升工程示范点，用好扶贫专项资金，大力推进贫困村道路、水利、电力、电子商务、公共服务等设施建设，补齐贫困地区基础设施和公共服务短板，提升贫困地区稳定脱贫能力。统筹县级扶贫资源，逐步推进有贫困人口的非贫困村通硬化路。加快易地扶贫搬迁项目建设，确保搬迁户搬得出、稳得住、有保障、能致富。

精准抓好扶志扶智。大力开展扶贫扶志专项行动，着力激发脱贫内生动力，充分调动贫困群众发展生产、脱贫致富的主动性，破除“等、靠、要”思想。大力推进技能脱贫，统筹整合培训资源，组织贫困家庭劳动力参加各类技能培训，开展贫困家庭“两后生”职业培训，促进贫困劳动力就近就地实现就业，提升贫困人口脱贫致富能力。加大无劳动能力贫困户政策兜底保障力度。

（四）在产业高质量发展上实现突破。深入供给侧结构性改革，贯彻落实“巩固、增强、提升、畅通”八字方针，加快产业转型升级步伐，全面推动产业高质量发展。

加快培育四大千亿产业。深入实施广西先进装备制造城（玉林）五年行动计划，推进玉柴“二次创业”，做强内燃机板块，打造新能源汽车板块，推动智能制造创新发展，培育军民融合产业基地，打造千亿机械制造产业。加快玉林（福绵）节能环保产业园和生态纺织服装产业园、玉林（博白）节能环保产业园、陆川纺织服装产业园、北流服装皮革产业园建设，补强纺织产业板块，促进印染企业全部退城进园，加快服装皮革产业高端化发展，打造千亿服装产业。加快建设龙潭产业园、北流帝森绿色科技创业园、玉林（兴业）新材料生态产业园，规划建设轻工产业园和精细化工产业园，大力发展不锈钢全产业链、锂电池新材料、精细化工、碳酸钙上下游产业板块，打造千亿新材料产业。推动中药材专业市场转型升级，加快中医药健康产业园建设，延伸医药、食品产业链，打造千亿大健康产业。加快装配式建筑与现代绿色建材产业基地建设，大力发展装配式建材、钢架结构等新型建材产业。创新园区投融资、建设、招商机制，加快广西生态板材家具产业园等园区发展，做大做强陶瓷、林产化工、电子信息、铁锅、芒编等县域优势传统产业。

深入实施创新驱动发展战略。推动产学研协同创新，提高科技创新能力。加快玉林高新区建设，重点发展新一代电子信息、新材料、生物等新兴产业，打造区域性高新技术产业集聚区、产城融合示范区和创新人才高地。强化内燃机国家重点实验室建设，规划筹建军民融合先进机械装备研究院。加快发展企业研发机构，组建市级工程技术研究中心、企业技术中心8家。支持三环陶瓷研究所建设发展。加快市中小企业科技创新孵化中心建设，加大科技型企业培育力度，力争新增市级科技型中小企业10家。组织开展科技研发攻关，推动汽车制动毂自动化、挖掘机智能化等一批关键共性技术突破和集成应用。推进科技成果转化大行动，力争转化科技成果31项以上。加快智慧城市建设，积极发展物联网、云计算、大数据、北斗导航应用产业。积极培育发展节能环保、新能源、新能源汽车等战略性新兴产业，加快形成发展新动能。深入实施质量强玉和品牌发展战略，提升质量水平，打造“玉林制造”品牌。

推动现代服务业集聚发展。积极探索“互联网+”“文化+”产业新模式，大力培育电子商务、文化旅游、创意文化、数字经济、分享经济等新型服务业态。抓好39个重大旅游项目建设，大力开展4A级景区、旅游度假区、生态旅游示范区、星级乡村旅游区和农家乐创建工作，推动容县、北流创建国家全域旅游示范区，玉州、陆川创建自治区全域旅游示范区，打响“五彩玉林·田园都市”品牌。改造提升商贸物流业，加快推进万达广场、金城广场、奥园广场、万花楼、汇金广场、北流荣顾购物公园等重点商贸项目，形成一批城市核心商圈。培育壮大银丰国际中药港、毅德国际商贸城、宏进农产品批发市场、桂龙翔物流产业园等专业市场，新建改建一批农贸市场。大力发展冷链物流，加快冷库仓储项目建设。大力发展会展经济，办好玉博会、药博会，支持县（市、区）办好特色展会。积极开展“双百”促消费活动，打造“岭南都会”消费购物节品牌，多渠道促进城乡居民消费。

加快创建民营经济先行示范市。创新支持民营经济发展体制机制，推动制度创新、科技创新、模式创新和服务创新，创建一批民营经济示范县（市、区）、示范园区、示范乡镇。实施领军型企业、科技型中小企业、成长型企业、瞪羚企业等培育计划，着力培育一批民营经济骨干企业，力争新增规模以上工业企业30 家。鼓励民营企业参与国企改革，进入基础设施、教育、文化、健康养老等领域。积极拓宽民营企业融资渠道，完善信贷融资、债券发行、基金、政府性融资担保等融资方式，优化“惠企贷”服务，合力化解民营企业融资难、融资贵问题。落实领导干部服务企业制度，加大对民营企业的帮扶力度。依法保护民营企业和企业家合法权益，保证企业依法平等使用生产要素、公平参与市场竞争。深入实施“企业家培养工程”，培养一批懂技术、会管理、善营销的民营企业家。加快玉商大厦建设，促进民营企业家交流合作。

（五）在项目建设上实现突破。坚持把项目建设作为稳增长强后劲的重要抓手，着力破解项目建设瓶颈，大力推进一批重大项目，以项目大建设促进玉林大发展。

强化项目用地保障。大力实施一批“旱改水”、耕地开垦项目，用好城乡建设用地增减挂钩等土地政策，继续实行市级补充耕地指标交易，破解耕地占补平衡瓶颈。创新土地利用方式，新增建设用地优先保障重大项目，小项目主要利用闲置土地，推广多层厂房，提高土地利用效率。开展闲置土地处置攻坚行动，清理批而未供、供而未用的土地，推进工业用地二次开发，加快盘活存量土地。创新征地拆迁工作机制和补偿机制，探索多种形式的安置方式，着力破解安置难、成本高的问题，确保净地供应。

全力推动项目落地做成。深入开展“重大项目建设攻坚突破年”活动，进一步完善重大项目推进机制、领导挂钩项目制度，加快推进一批重大项目建设，今年计划新开工项目280个，总投资2027亿元；续建项目278个，总投资1861亿元；竣工项目105个，总投资206亿元。重点推进柳州医药、亚邦汽配等项目开工建设，加快推进玉柴新能源汽车、南方黑芝麻容县新厂、五丰机械新生产基地等在建项目，力争长源东谷气缸体、3D打印、金特安特种轮胎、康臣玉药、睿盛农牧产品深加工等项目竣工。做好玉林现代化农产品深加工生产线、玉林航空智能制造产业化、新希望养殖及深加工等一批项目前期工作。积极争取中央投资项目，力争中央预算内投资完成6亿元以上。引导支持民营资本投向基础设施和产业项目，力争民间投资增长11%以上。

加大招商引资力度。深入开展“产业大招商攻坚突破年”活动，深化招商引资体制机制改革，实行“一把手”招商工作机制，完善考核激励、委托招商、项目落地、“保姆式”服务等制度。创新招商引资方式，建立专业招商团队，建立健全产业链招商、资本招商、园区招商、集群招商等专业化招商机制，实现招大引强的目标。推动“玉商回归”“侨商回归”，力争引回更多人才、项目、资金、技术。坚持开展“每月一专题、每季一活动”精准招商，着力引进一批龙头型、基地型、科技型重大项目。抓好正威国际、宝能集团、正大集团、卡为集团、深圳欣旺达等企业项目签约落地。

（六）在改革开放上实现突破。站在改革开放40周年新起点上再出发，以敢闯敢试、敢为人先的胆识气魄，坚定不移深化改革开放，努力开创玉林新一轮发展新局面。

全面优化营商环境。深入开展“优化营商环境攻坚突破年”活动，对标世界银行营商环境评价指标体系，深入实施优化营商环境大行动三年计划，进一步改善政务、建设经营、融资、税费、人力资源、通关、信用、法治等八大环境，营造最优发展环境。深化“放管服”改革、商事制度改革和投资项目审批制度改革，实现企业开办、不动产登记、投资项目等行政审批时限压缩一半。积极推动“互联网+政务服务”，加快政务服务“一张网”建设，大力推进“一事通办”，90%以上的行政审批事项和公共服务事项“最多跑一次”，甚至“一次都不用跑”。全面贯彻落实减税降费政策，多措并举降低企业成本，减轻企业负担。规范政商交往行为，构建“亲”“清”新型政商关系。加快社会信用体系建设，加强重点领域信用联合惩戒，形成诚信守法的长效机制。

深化重点领域改革。积极稳妥推进机构改革，3月底基本调整到位。深化农村改革，抓好我市全国农村改革试验区各项改革任务，推进北流市农村集体经营性建设用地入市、土地征收和农村宅基地三项制度改革。深化国企国资改革，继续加快政府经营性公司资源整合步伐，积极组建水务集团、建工集团和公交集团，做大做强做优玉林城投集团、玉林投资集团和玉林交旅集团等企业。深化财政管理改革，科学划分市与城区的财权、事权责任。深化金融改革，推进博白县农村信用社改制。充分发挥玉林金融投资集团、玉林市农业信贷担保公司的作用，服务实体经济发展。深化城市管理体制改革，推动城市管理重心下移，解决执法职责交叉、城市管理效能不高问题。

大力推进向海发展。加快“东融”步伐，加强与粤港澳大湾区产业合作，积极承接东部沿海地区新一轮产业转移。加快“南向”步伐，推进沙河至铁山港东岸货运专线铁路项目，加快龙港新区龙潭产业园、玉港合作园建设，大力推进柳钢中金500万吨不锈钢基地、银亿红土镍矿资源绿色开发综合利用等重大项目，构建新材料、再生资源环保、临港经济等三大产业发展格局。坚定不移融入“一带一路”建设，推进中国—文莱（玉林）健康产业园、中泰（玉林）国际旅游文化产业园等重大项目建设。实施“走出去”三年行动计划，服务好仲礼瓷业马来西亚项目、瑞安文莱香料加工与贸易项目、三环陶瓷迪拜项目建设。

（七）在城市建设管理上实现突破。深入实施大城市战略，突出规划引领，强化基础支撑，提升城市管理水平和功能品位，加快打造区域性大城市。

提升中心城市辐射带动能力。加快高铁新城、五彩田园、会仙河公园和北流家具大街片区建设，推动公共交通、信息服务、基础设施无缝对接，促进玉林北流同城化发展。加快城北中央商务区、玉东湖、玉西湖、玉东文体路生态城、广西医科大学玉林校区片区、龟山大道片区开发，促进城市多点布局协同发展。加强城市立体交通规划，加快二环南路扩建、二环西路、民主南路、园博园至五彩田园公路、江南路延长线、人民东路人行天桥等项目建设，统筹推进城市道路“白改黑”，进一步完善城市路网。加快旧城改造，推进棚户区改造项目建设。加快海绵城市建设，积极推进二环东路、二环南路地下综合管廊建设，完善城市供水污水管网、公厕、环卫、停车场等公共设施，增强城市配套功能。加快城市广场、公园绿地、城区风貌改造等市政项目建设，不断提升城市形象。

提升城市管理水平。深入推进“四城同创”，推动人居环境和社会文明程度整体提升。创新城市管理方式，构建数字化城市管理平台，实现科学化、精细化、智能化、法治化管理。全面加强城市环卫保洁工作，增加环卫设施投入，落实门前“三包”制度、24小时保洁制度，推进城区生活垃圾分类收集和处置工作，规划建设建筑垃圾消纳项目。加大城市管理执法力度，深入开展城市交通秩序、市容环境、农贸市场、房屋租赁专项整治，全面治理车辆乱停乱放、乱摆乱卖、占道经营等行为。继续抓好城区小街小巷“净化、美化、亮化、硬化”工作。加快发展城市公共交通，推进智慧交通建设，提升市民出行便捷度。

构建现代化城镇体系。统筹城乡规划布局，优化城镇空间格局，推动中心城市与县域协同发展。推进县城有序开发建设，提高县域城镇化水平。支持县域开展创城创建活动，提升县城知名度和美誉度。积极推进具备条件的地区撤县设市。坚持提质扩容和凸显特色并重， 培育一批经济强镇、商贸重镇、文化古镇、旅游名镇。推进玉州中医药文化小镇、玉东健康小镇、北流陶瓷小镇、福绵服装小镇、容县沙田柚小镇、博白客家书香小镇、陆川名猪小镇、兴业运动小镇等特色小镇建设，增强县域发展新动能。

（八）在乡村振兴上实现突破。深入实施乡村振兴战略，加快推进农业农村现代化，促进农业增效、农民增收、乡村生态宜居，争当全区乡村振兴的示范市和排头兵。

大力发展现代特色高效农业。建设一批粮食绿色高产高效全程机械化示范基地，提高粮食生产水平。实施现代特色农业产业“10+3”提升行动，做强做优容县沙田柚、北流荔枝、陆川橘红、博白桂圆肉、玉州仁东大蒜、福绵八角、兴业粉蕉、玉林三黄鸡等特色优势产业。实施农业绿色品牌战略，大力培育“三品一标”农产品品牌和生态原产地保护产品品牌。深入推进现代特色农业示范区建设增点扩面提质升级三年行动，建成自治区级核心示范区3个以上、县级示范区7个以上、镇级示范园80个以上和一批村级示范点。加快现代农业产业园、科技园和田园综合体建设，争创一批国家级、自治区级特色农产品优势区。加强动物防疫体系建设，促进畜牧业健康发展。推动农村一二三产业融合发展，加快兴业县中农联农产品电商物流项目、兴业县金怡百万头生猪深加工项目、中国—东盟农产品深加工产业园等项目建设，做大做强农产品加工业。

加强农村基础设施建设。深入实施乡村振兴基础设施公共服务能力提升三年行动计划。抓好“五小水利”工程建设，加快秦镜水库建设进度，做好龙云灌区工程和蕉林水库、黄章水库、六林水库前期工作。深入实施农村饮水安全巩固提升工程，实现农村集中式饮水人口比例达到83％以上。推进农村电网新一轮改造升级，提高供电能力。实施信息进村入户工程，加快光纤宽带网络和无线网络建设。

深入推进幸福乡村建设。实施乡村风貌提升三年行动，推进美丽宜居试点镇、乡土特色试点村建设。深入开展农村人居环境整治三年行动，推进农村生活垃圾、农村厕所无害化治理，完善农村污水处理设施，深化农村环境集中连片综合治理，改善农村人居环境。抓好乡村精神文明建设，大力发展乡村文化产业。加快推进乡村治理体系和治理能力现代化，健全农村社会矛盾纠纷化解机制，依法严厉打击危害农村稳定、破坏农业生产和侵害农民利益的违法犯罪活动，维护农村和谐稳定。

（九）在民生改善上实现突破。坚持以人民为中心的发展思想，集中力量抓好社保、健康、教育、水利、安居、农补、生态、文化、扶贫、科技等十大类惠民项目，补齐民生短板，加强民生保障，让人民群众的生活更加幸福美好。

加快发展教育事业。继续推进学校建设提升工程，开工建设学校16所、续建37所。深入实施第三期学前行动计划，推进市第二、第三幼儿园等城区幼儿园建设。加快玉州教育城、秀石路中学等学校建设，缓解玉林城区“大班额”“上学难”问题。加快义务教育均衡发展，确保博白县通过国家评估认定、福绵区通过自治区督导评估。深入实施高中阶段教育普及攻坚计划，加快市一中新校区、福绵高中迁建、市十中改扩建等项目建设，启动高考综合改革工作。加快发展现代职业教育，开工建设职业教育园区（一期），推进博白县职业学校迁建。支持玉林师院申硕攻坚工作。加快筹建玉林职业技术学院。实施第二期特殊教育提升计划，推进特殊教育学校建设。支持发展民办教育，加快育林中学等学校建设。加强师德师风建设，培养高素质教师队伍。深化教育综合评价改革，促进学生德、智、体、美、劳全面发展。实施全民科学素质行动计划纲要，提升全民科学素养。

加强就业和社会保障工作。把稳就业摆在突出位置，落实新一轮就业创业政策，重点做好高校毕业生、城镇就业困难人员、农村转移劳动力、退役军人等各类群体就业工作；推进国家创业担保贷款工作，加强创业孵化基地、农民工创业园建设，扶持各类群体创业就业，实现城镇新增就业3万人。继续加大农民工工资治欠保支工作力度。全面实施全民参保计划，加大社保扩面征缴力度，继续阶段性降低社保缴费费率。实施职工大额补充医疗保险制度。完善社会保障卡制度。创新住房公积金管理，打造智慧公积金“玉林模式”。加快保障性住房建设，新开工建设棚户区改造6000套以上。加快市颐养园（二期）、市益寿园搬迁项目建设，积极发展养老服务业，推进政府购买居家养老服务。加快市军供站项目建设，规划建设市流浪乞讨人员安置托养中心。加大残疾人权益保障力度。继续关爱“三留守”人员。深入实施中长期青年发展规划，加强未成年人保护工作。深入实施妇女儿童“两个规划”，促进妇女儿童事业全面发展。

繁荣发展文化广播电视体育事业。实施艺术创作“十百千”计划，创作一批戏剧、音乐、舞蹈、小品、曲艺、杂技等剧目。巩固提升“国家公共文化服务体系示范区”创建成果，投入使用市图书馆新馆，加快市工人文化宫、市群众艺术馆新馆、市档案馆新馆、市科技馆、王力博物馆、广西华侨博物馆、博白博物馆等项目规划建设，支持玉林师范学院桂东南历史文化博物馆建设。建设市级非物质文化遗产生产性保护基地。实施文化惠民富民工程，推进全民阅读，打造“书香玉林”。大力发展广播电视事业，实施数字玉林“广电云”村村通户户用工程。推进媒体融合发展，加快县级融媒体中心建设。加快推进第二轮地方志编修工作。广泛开展全民健身活动，组织开展第十一届广西体育节·玉林市全民健身系列活动，筹办第二届北部湾城市运动会，开工建设威壮体育公园。

推进健康玉林建设。深入实施全民健康素养促进行动计划，开展健康科普宣传，加强社会心理健康服务，引导群众坚持健康生活方式。加大社区、镇村卫生服务机构标准化建设力度，不断增强医疗卫生公共服务能力。深化医药卫生体制改革和公立医院改革，加快紧密型医疗联合体建设，推进分级诊疗更加惠及百姓。深化医疗服务价格、医保支付方式和公立医院人事薪酬制度等方面改革，持续改善医疗服务质量，努力缓解群众“看病难、看病贵”问题。实施健康扶贫工程，逐步解决因病致贫、因病返贫问题。坚持中西医并重，推动中医药、民族医药发展。支持社会办医。发展健康服务产业。加快广西医科大学玉林校区（二期）建设，实现今年秋季期本科、大专招生办学。

完善社会治理体系。落实防范责任，进一步防范化解政府债务风险。持续打击非法金融活动，防范区域性系统性金融风险。建立健全城乡居民参与社区治理、基层公共事务和公益事业的体制机制，提升城乡社区治理水平。深入开展“七五”普法，健全公共法律服务体系，加强法律援助工作。完善预防和化解社会矛盾机制，推进信访工作“三化”建设。完善立体化社会治安防控体系，深入开展扫黑除恶专项斗争和禁毒严打整治专项行动，深挖“保护伞”，严打涉枪涉爆、涉黄涉赌、跨区域职业犯罪和电信网络诈骗等各类违法犯罪活动，加强社区矫正工作，全力维护良好治安。持续开展安全生产大排查、大整治行动，推进企业安全生产标准化建设，严密防范重特大安全生产事故。深入开展食品药品安全隐患排查整治行动，努力让群众吃得放心、吃得安全。加强餐厨剩余物监管和处理。深入开展成品油市场整治，严厉打击非法经营成品油行为。加强消防基础设施建设，强化消防宣传教育。抓好森林防火工作。加强地质灾害防治工作。做好地震灾害预防工作，推进地震应急救援训练基地建设。完善公共安全预警、应急、救援体系，提高预防和处置突发事件能力。加强民族团结教育，筑牢人民群众的中华民族共同体意识。依法管理宗教事务。积极推进人民防空防护体系建设。加强国家安全教育，强化国家安全工作责任落实。加强国防教育，强化国防动员和后备力量建设，深化双拥共建工作，争创“全国双拥模范城”，深入推进军民融合发展。

四、加强政府自身建设

进入新时代，肩负新使命，踏上新征程，对政府推进发展、服务群众提出了新的更高要求。我们要顺应人民群众新期待，全面加强政府自身建设，敢于担当，勤勉敬业，脚踏实地，善作善成，不负人民重托，当好新时代答卷人。

（一）全面加强政治建设。深入学习贯彻习近平新时代中国特色社会主义思想，自觉增强“四个意识”，坚定“四个自信”，坚决做到“两个维护”，在政治立场、政治方向、政治原则、政治道路上同党中央保持高度一致。坚持把旗帜鲜明讲政治贯穿于政府工作全过程、各方面，坚决执行党的理论路线方针政策，坚决贯彻落实中央、自治区和市委决策部署，坚持向市委报告工作，切实做到信念过硬、政治过硬、责任过硬、能力过硬、作风过硬。

（二）深入推进依法行政。加快建设法治政府，坚持带头学法、模范守法、善于用法、秉公执法。自觉接受市人大及其常委会的法律监督和工作监督，主动接受市政协民主监督，以及监察监督、司法监督、群众监督和舆论监督，广泛听取各民主党派、工商联、无党派人士和广大群众意见，抓好人大代表建议和政协委员提案办理。完善重大行政决策程序制度、合法性审查制度、终身责任追究制度和倒查机制，提高行政决策科学化、民主化、法治化水平。加强规范性文件出台审查，积极构建公平竞争的市场体系。大力推行政务公开，让权力在阳光下运行。

（三）切实加强作风建设。开展“解放思想再出发，激励干部敢为先勇担当善作为”活动，着力破除与新时代要求不相适应的思想观念和思维定势。大力倡导“担当为要、实干为本、发展为重、奋斗为荣”的为政理念，传承弘扬改革精神，激励广大干部新时代新担当新作为。严格政府绩效管理，建立健全正向激励和容错纠错机制，让想干事、能干事、干成事的干部受到激励和表彰。改进和规范政务督查，继续抓好国务院第五次大督查移交问题整改落实。加强政风监督，驰而不息整治“四风”，力戒形式主义、官僚主义，坚决把中央八项规定精神落到实处。

（四）始终保持清正廉洁。落实全面从严治党主体责任和“一岗双责”，严格执行党内政治生活若干准则、廉洁自律准则、问责条例，推动政府系统党风廉政建设和反腐败工作向纵深发展。加大审计监督力度，严管公共资金、公共资源交易、国有资产资本，严格控制“三公”经费和一般性支出。坚持惩防并举，大力支持配合纪委监委工作，紧盯重大工程、重点领域、关键岗位，持续整治群众身边腐败和作风问题，巩固发展反腐败斗争压倒性胜利，一体推进不敢腐、不能腐、不想腐，让政府工作人员知敬畏、存戒惧、守底线，永葆为民务实清廉的政治本色。

各位代表，惟其艰难，方显勇毅；惟其磨砺，始得玉成。让我们更加紧密团结在以习近平同志为核心的党中央周围，在市委的坚强领导下，以坚如磐石的信心、只争朝夕的劲头、坚忍不拔的毅力，一往无前、永不停步，在“建设壮美广西、共圆复兴梦想”的新征程中展现新作为、创造新业绩，奋力谱写新时代玉林发展新篇章，以优异成绩向新中国成立70周年献礼！
